# Supplementary material for: “I feel good… I knew that I would…”: The role of self in musical reward across cultures
Source: PLoS One. 2026 Jan 6;21(1):e0340597. doi: 10.1371/journal.pone.0340597 (PMC12774338; doi:10.1371/journal.pone.0340597)
Supplement: S1 Appendix — (DOCX) [file pone.0340597.s001.docx]

**Supporting Materials**

**Appendix A – Participant Characteristics**

Table 1a

*Participants’ Nationality*

| N/S American  (*n* = 255) | | European  (*n* = 103) | | Asian  (*n* = 76) | |
| --- | --- | --- | --- | --- | --- |
| American | 214 (49.3%) | British | 73 (16.8%) | Chinese | 20 (4.6%) |
| Brazilian | 2 (0.5%) | Dutch | 1 (0.2%) | Dual Nationality | 7 (1.6%) |
| Canadian | 38 (8.8%) | English | 10 (2.3%) | Filipino | 3 (0.7%) |
| Jamaican | 1 (0.2%) | European | 1 (0.2%) | Hmong | 1 (0.2%) |
|  |  | German | 2 (0.5%) | Hong Kong | 2 (0.5%) |
|  |  | Hungarian | 1 (0.2%) | Indian | 8 (1.8%) |
|  |  | Irish | 1 (0.2%) | Indonesian | 3 (0.7%) |
|  |  | Italian | 1 (0.2%) | Korean | 1 (0.2%) |
|  |  | Polish | 2 (0.5%) | Laotian | 1 (0.2%) |
|  |  | Romanian | 1 (0.2%) | Malaysian | 1 (0.2%) |
|  |  | Scottish | 3 (0.7%) | Nepali | 3 (0.7%) |
|  |  | Spanish | 3 (0.7%) | Singaporean | 22 (5.1%) |
|  |  | Ukrainian | 1 (0.2%) | Taiwanese | 2 (0.5%) |
|  |  | Welsh | 1 (0.2%) | Thai | 1 (0.2%) |
|  |  |  |  | Vietnamese | 1 (0.2%) |

Table 1b

*Participants’ Country of Residence*

| North America  (*n* = 281) | | UK/Europe  (*n* = 119) | | Asia  (*n* = 34) | |
| --- | --- | --- | --- | --- | --- |
| Canada | 45 (10.4%) | England | 43 (9.9%) | China | 10 (2.3%) |
| U.S. | 236 (54.4%) | France | 1 (0.2%) | India | 1 (0.2%) |
|  |  | Ireland | 1 (0.2%) | Singapore | 22 (5.1%) |
|  |  | Scotland | 4 (0.9%) | Taiwan | 1 (0.2%) |
|  |  | U.K. | 70 (16.1%) |  |  |

Table 1c

*Participants’ Ethnicity*

| White/Caucasian  (*n* = 251) | | Black, Hispanic, & Mixed  (*n* = 53) | | Asian or Pacific Islander  (*n* = 130) | |
| --- | --- | --- | --- | --- | --- |
| Albanian | 1 (0.2%) | Aboriginal | 1 (0.2%) | Asian | 58 (13.4%) |
| White | 249 (57.4%) | Black | 24 (5.5%) | Bangladeshi | 1 (0.2%) |
| White Gypsy | 1 (0.2%) | Egyptian | 2 (0.5%) | Bengali | 2 (0.5%) |
|  |  | Hispanic | 2 (0.5%) | Chinese | 42 (9.7%) |
|  |  | Latinx | 2 (0.5%) | Filipino | 6 (1.4%) |
|  |  | Mixed Race | 22 (5.1%) | Indian | 10 (2.3%) |
|  |  |  |  | Japanese | 4 (0.9%) |
|  |  |  |  | Korean | 3 (0.7%) |
|  |  |  |  | Malay | 2 (0.5%) |
|  |  |  |  | Native Hawaiian | 1 (0.2%) |
|  |  |  |  | Southeast Asian | 1 (0.2%) |
