# Supplementary material for: “I feel good… I knew that I would…”: The role of self in musical reward across cultures
Source: PLoS One. 2026 Jan 6;21(1):e0340597. doi: 10.1371/journal.pone.0340597 (PMC12774338; doi:10.1371/journal.pone.0340597)
Supplement: S2 Appendix — (DOCX) [file pone.0340597.s002.docx]

**Supporting Materials**

**Appendix B – Full Multiple Regression Analysis Results**

Table 2

*Results for Musical Reward Using Yang’s Self-Construal Scale (Y-SCS) as Predictors*

|  | Outcome Variable | | | | |
| --- | --- | --- | --- | --- | --- |
| Predictor | *B* | *SE* | *β* | *p* | *sr^2^* |
|  | Social Reward | | | | |
| Difference vs. similar to others | 0.06 | 0.04 | 0.07 | .177 | .063 |
| **Self-containment vs. connectedness to others** | **-0.16** | **0.04** | **-0.24** | **.000** | **-.207** |
| Self-direction vs. reception to influence | -0.01 | 0.05 | -0.01 | .926 | -.004 |
| Self-expression vs. harmony | 0.04 | 0.04 | 0.05 | .408 | .038 |
| Consistency vs. variability | 0.07 | 0.03 | 0.13 | .014 | .114 |
| Decontextualized vs. contextualized self | -0.03 | 0.04 | -0.05 | .354 | -.043 |
| **Self-reliance vs. dependence on others** | **-0.10** | **0.04** | **-0.15** | **.007** | **-.125** |
| Self-interest vs. commitment to others | 0.05 | 0.04 | 0.07 | .223 | .056 |
|  | Musical Seeking | | | | |
| **Difference vs. similar to others** | **0.10** | **0.04** | **0.15** | **.005** | **.130** |
| **Self-containment vs. connectedness to others** | **-0.15** | **0.03** | **-0.24** | **.000** | **-.213** |
| Self-direction vs. reception to influence | -0.02 | 0.05 | -0.03 | .707 | -.017 |
| Self-expression vs. harmony | 0.06 | 0.04 | 0.10 | .096 | .077 |
| Consistency vs. variability | 0.05 | 0.03 | 0.11 | .035 | .097 |
| Decontextualized vs. contextualized self | -0.06 | 0.03 | -0.10 | .060 | -.087 |
| Self-reliance vs. dependence on others | -0.01 | 0.03 | -0.01 | .883 | -.007 |
| Self-interest vs. commitment to others | 0.09 | 0.04 | 0.13 | .019 | .108 |
|  | Emotion Evocation | | | | |
| **Difference vs. similar to others** | **0.10** | **0.04** | **0.16** | **.004** | **.135** |
| Self-containment vs. connectedness to others | -0.06 | 0.03 | -0.10 | .053 | -.091 |
| Self-direction vs. reception to influence | 0.06 | 0.04 | 0.10 | .155 | .067 |
| Self-expression vs. harmony | 0.04 | 0.04 | 0.07 | .266 | .052 |
| Consistency vs. variability | -0.05 | 0.02 | -0.12 | .031 | -.101 |
| Decontextualized vs. contextualized self | -0.02 | 0.03 | -0.03 | .594 | -.025 |
| Self-reliance vs. dependence on others | -0.04 | 0.03 | -0.07 | .253 | -.054 |
| Self-interest vs. commitment to others | -0.07 | 0.04 | -0.11 | .050 | -.092 |
|  | Mood Regulation | | | | |
| Difference vs. similar to others | 0.05 | 0.03 | 0.09 | .115 | .073 |
| **Self-containment vs. connectedness to others** | **-0.13** | **0.03** | **-0.27** | **.000** | **-.236** |
| Self-direction vs. reception to influence | 0.05 | 0.04 | 0.09 | .177 | .062 |
| Self-expression vs. harmony | 0.04 | 0.03 | 0.08 | .187 | .064 |
| Consistency vs. variability | -0.00 | 0.02 | -0.01 | .831 | -.010 |
| Decontextualized vs. contextualized self | 0.00 | 0.03 | 0.01 | .916 | .005 |
| Self-reliance vs. dependence on others | 0.03 | 0.03 | 0.07 | .220 | .059 |
| Self-interest vs. commitment to others | 0.02 | 0.03 | 0.03 | .550 | .029 |
|  | Sensory-Motor | | | | |
| Difference vs. similar to others | 0.07 | 0.04 | 0.10 | .083 | .080 |
| **Self-containment vs. connectedness to others** | **-0.18** | **0.04** | **-0.26** | **.000** | **-.229** |
| Self-direction vs. reception to influence | 0.10 | 0.05 | 0.13 | .058 | .087 |
| Self-expression vs. harmony | 0.04 | 0.05 | 0.05 | .401 | .039 |
| Consistency vs. variability | 0.04 | 0.03 | 0.08 | .133 | .069 |
| Decontextualized vs. contextualized self | 0.01 | 0.04 | 0.01 | .812 | .011 |
| Self-reliance vs. dependence on others | -0.03 | 0.04 | -0.05 | .387 | -.040 |
| Self-interest vs. commitment to others | -0.01 | 0.04 | -0.01 | .832 | -.010 |

*Note.* *B*, unstandardized beta; *β*, standardized beta; *sr^2^*, squared semi-partial (or part) correlations. Positive beta coefficients indicate a positive association with the independent pole of the Y-SCS factor whereas negative beta coefficients indicate a positive association with the interdependent pole of the Y-SCS factor. Bold values indicate statistical significance (using an *α* of .01).
