# Supplementary material for: “I feel good… I knew that I would…”: The role of self in musical reward across cultures
Source: PLoS One. 2026 Jan 6;21(1):e0340597. doi: 10.1371/journal.pone.0340597 (PMC12774338; doi:10.1371/journal.pone.0340597)
Supplement: Attachment — Submitted filename: ReviewerResponse_20250701.docx [file pone.0340597.s003.docx]

| **Academic Editor’s Comments** | **Author Response** |
| --- | --- |
| Journal requirements:  When submitting your revision, we need you to address these additional requirements.   1. Please ensure that your manuscript meets PLOS ONE's style requirements, including those for file naming. The PLOS ONE style templates can be found at   [https://journals.plos.org/plosone/s/file?id=wjVg/PLOSOne_formatting_sample_main_body.pdf](https://track.editorialmanager.com/CL0/https:%2F%2Fjournals.plos.org%2Fplosone%2Fs%2Ffile%3Fid=wjVg%2FPLOSOne_formatting_sample_main_body.pdf/1/010f01972f9881e1-c7332929-1571-4218-9ab0-0726d50bf1e2-000000/x6TKOiaL4W_Sc__JWg8LztNbyIjWhWMEZ-CFgj7D62s=213)  and  [https://journals.plos.org/plosone/s/file?id=ba62/PLOSOne_formatting_sample_title_authors_affiliations.pdf](https://track.editorialmanager.com/CL0/https:%2F%2Fjournals.plos.org%2Fplosone%2Fs%2Ffile%3Fid=ba62%2FPLOSOne_formatting_sample_title_authors_affiliations.pdf/1/010f01972f9881e1-c7332929-1571-4218-9ab0-0726d50bf1e2-000000/Ve7fs_QGS_qvoLOKsuZQIy4Q5rwpNRJSt-lgeBGsacs=213) | I have edited the manuscript to be consistent with the PLOS ONE’s style requirements. |
| 2. Thank you for stating the following financial disclosure:   “This work was supported by the Arts & Humanities Research Council (grant number AH/R012733/1) through the White Rose College of the Arts & Humanities.”  Please state what role the funders took in the study.  If the funders had no role, please state: "The funders had no role in study design, data collection and analysis, decision to publish, or preparation of the manuscript."  If this statement is not correct you must amend it as needed.  Please include this amended Role of Funder statement in your cover letter; we will change the online submission form on your behalf. | The funders had no role. I have amended this in my cover letter. |
| 3. Thank you for stating the following in the Acknowledgments Section of your manuscript:   “I would like to thank Nikki Dibben and Renee Timmers for their feedback on earlier versions of this article. This work was supported by the Arts & Humanities Research Council (grant number AH/R012733/1) through the White Rose College of the Arts & Humanities.”  We note that you have provided funding information that is currently declared in your Funding Statement. However, funding information should not appear in the Acknowledgments section or other areas of your manuscript. We will only publish funding information present in the Funding Statement section of the online submission form.  Please remove any funding-related text from the manuscript and let us know how you would like to update your Funding Statement. Currently, your Funding Statement reads as follows:  “This work was supported by the Arts & Humanities Research Council (grant number AH/R012733/1) through the White Rose College of the Arts & Humanities.”  Please include your amended statements within your cover letter; we will change the online submission form on your behalf. | I have removed the funding information from my acknowledgements. I have updated my cover letter accordingly. |
| 4. When completing the data availability statement of the submission form, you indicated that you will make your data available on acceptance. We strongly recommend all authors decide on a data sharing plan before acceptance, as the process can be lengthy and hold up publication timelines. Please note that, though access restrictions are acceptable now, your entire data will need to be made freely accessible if your manuscript is accepted for publication. This policy applies to all data except where public deposition would breach compliance with the protocol approved by your research ethics board. If you are unable to adhere to our open data policy, please kindly revise your statement to explain your reasoning and we will seek the editor's input on an exemption. Please be assured that, once you have provided your new statement, the assessment of your exemption will not hold up the peer review process. | Noted. My entire data will be made freely accessible if my manuscript is accepted for publication. I have updated my data availability statement accordingly on the submission form. |
| 5. Please include captions for your Supporting Information files at the end of your manuscript, and update any in-text citations to match accordingly. Please see our Supporting Information guidelines for more information: <http://journals.plos.org/plosone/s/supporting-information>. | I have included the captions for my Supporting Information files at the end of the manuscript. I have also updated the in-text citations to match accordingly. |
| Additional Editor Comments:  Both MANCOVA (page 11) and multiple regressions (page 12) need to be controlled for the inlfated Type-I error. PLOS ONE accepts manuscripts not based on the impacts of findgins, but the rigor of methods. Currently, the familiy-wise Type-I error appears largely inflated. | For MANCOVA, I believe there is a higher risk of Type-II error (rather than Type-I error) given that I have included several covariates. So, I have left it as is.  Thank you for the suggestion for controlling for Type-I error for the multiple linear regressions. I have applied a Bonferroni correction and edited my results/discussion sections accordingly. |
