# Supplementary material for: “I feel good… I knew that I would…”: The role of self in musical reward across cultures
Source: PLoS One. 2026 Jan 6;21(1):e0340597. doi: 10.1371/journal.pone.0340597 (PMC12774338; doi:10.1371/journal.pone.0340597)
Supplement: Attachment — Submitted filename: ReviewerResponse_20251001.docx [file pone.0340597.s004.docx]

| **Additional Editor Comments** | **Author Response** |
| --- | --- |
| In particular, the current study partly relies on MTurk participants, whose validity has been questioned and for whom many pitfalls have been discussed. See <https://doi.org/10.1371/journal.pone.0057410> and <https://doi.org/10.1146/annurev-clinpsy-021815-093623> for reference. Please discuss the potential limitations of this sample when interpreting the present results. | Thank you for this suggestion. I incorporated this into my limitations on p. 21. |

| **Reviewer’s Comments** | **Author Response** |
| --- | --- |
| 1. General Assessment The manuscript addresses a timely and potentially valuable topic related to personality traits, self-construals, and collectivism-individualism distinctions within a cross-cultural psychological framework. The authors’ attempt to explore subtle dynamics between personality dimensions (as measured through the BFI) and cultural self-views (independent vs. interdependent self-construals) reflects a clear alignment with ongoing theoretical efforts in cultural psychology. The research is novel in its focus on linking self-construal types with Big Five dimensions within a specific cultural context, which is under-represented in the literature. The study is based on a reasonably large sample (N=435), and the statistical analyses are generally appropriate. However, there are substantial conceptual and methodological limitations that require thorough addressing before the manuscript can be considered for publication. | Thank you for taking the time to review my article and for the opportunity to edit my paper. I have considered your feedback and have incorporated them accordingly. Please see my responses to your comments below. |
| 2. Major Concerns 2.1. Grouping Procedure and Conceptual Validity A critical issue undermining the paper’s internal validity lies in the arbitrary dichotomization of participants into two groups – “collectivists” (n=73) and “individualists” (n=361). Aside from the numerical inconsistency (73 + 361 = 434, not 435 – this should be explicitly corrected), the categorization threshold appears both statistically and conceptually fragile. Someone scoring 49 is considered “collectivist”, while someone scoring 51 is “individualist”, although these individuals may be typologically indistinguishable. No buffer zone or standard deviation criterion was applied to ensure clear group separation, which significantly weakens the interpretation of group differences. I strongly recommend using a buffer zone (e.g., excluding cases close to the median, or applying a 0.5 SD band around the cut-off point) to enhance group distinctiveness – even at the cost of reducing subsample sizes. Without this, the derived typologies risk being statistically artificial and theoretically ambiguous, casting doubt over the discussion’s strength. | Thank you for this suggestion. Indeed, I considered various analytical options given the heterogeneity of the sample (see S1 Appendix A). Based on this feedback, I have opted for another strategy. Instead of applying a buffer zone, I chose to retain as much of the sample as possible by conducting a multilevel regression analysis, nesting participants within regions (p. 13). I grouped participants based on the cultural distance hypothesis (p. 11), which assumes that cultures are similar if they are geographically close to one another. |
| 2.2. Psychometric Weakness of the BFI The psychometric indicators reported for several BFI scales (e.g., Openness α = .26; Agreeableness α = .48; Conscientiousness α = .51) are unacceptably low for scientific reporting. While the BFI is a widely used tool, its use in the present study raises red flags due to these sub-threshold reliability scores. Even the most liberal acceptance levels for internal consistency require Cronbach’s α > .60 (ideally ≥ .70). Given that many of the conclusions rest on personality trait comparisons, the low reliability coefficients seriously undermine the robustness of findings. The authors should consider: • either excluding problematic subscales from analysis, • or using a validated alternative instrument with stronger psychometric support for their target population. | Thank you for your feedback. I have removed this subscale from analysis and in the paper. |
| 2.3. Theoretical Framing of Self-Construals The manuscript relies on a binary construal of independent vs. interdependent self, with strong associations to the individualism–collectivism dimension. However, recent advances (e.g., Vignoles et al., 2016) challenge this simplistic East–West dichotomy and instead advocate for multidimensional models of selfhood. These include domains such as: • self-direction vs. heteronomy, • differentiation vs. similarity, • self-containment vs. connectedness. I recommend the authors critically engage with such multifaceted frameworks of selfhood and reflect on their potential implications for interpretation of results. This would substantially improve the theoretical sophistication of the manuscript. | Thank you for this suggestion. I have incorporated an eight-dimensional model of selfhood in the article (p. 10) and analysis (pp. 14-16). At this stage, I acknowledged that my findings are exploratory and should be interpreted with caution (p. 19). |
| 2.4. Lack of Power Analysis and Uneven Group Sizes Another important omission is the lack of a prior statistical power analysis, which would have been critical given the asymmetric group sizes (n=73 vs. n=361). The rationale for this division is already questionable (see 2.1), but even assuming such a dichotomy is acceptable, the authors should have: • justified whether the smaller group size (n=73) provides adequate power to detect medium or small effects; • reported effect size sensitivity and alpha thresholds adopted in the study; • possibly explored balanced or bootstrapped re-sampling techniques to control for sample-size-induced bias. Given that all conclusions hinge on this between-group comparison, the current design may lead to both inflated Type I errors (due to multiple testing) and Type II errors (in the underpowered subgroup). The manuscript would greatly benefit from including: • a post hoc power analysis, at minimum, and preferably; • a justification for group sizes based on a priori power estimations aligned with expected effect sizes (e.g., based on literature or pilot studies). | Thank you for your comment. Given that this is the first study (to the best of my knowledge) to explore the relationship between self-construal and musical reward, I adopted an exploratory approach, striving for as large a sample as possible to see if a relationship exists. Thus, no a priori power analysis was analysed.  Given my new analytical approach, I did not report a post hoc power analysis as I did not compare two groups. I did report the post hoc power analysis for the multiple regression analysis involving the eight-dimensional model of self-construal (p. 15). |
| 3. Additional Issues and Clarifications • Statistical Validity & Type I Error: Regarding the editor's observation on Type I error inflation, the current analysis would indeed benefit from correction for multiple comparisons. Bonferroni correction, while conservative, is an acceptable first step to mitigate this issue. However, given the large number of comparisons, the authors might also consider: o Holm-Bonferroni (a sequentially rejective procedure), o or False Discovery Rate (FDR) methods (e.g., Benjamini–Hochberg), which preserve power while reducing false positives. Regardless of the method chosen, this correction must be transparently reported and integrated into result interpretation. • Clarification needed on the rationale for using two-sample t-tests instead of more robust multivariate techniques (e.g., MANOVA), given the multiple dependent variables and group comparisons. • Literature is overly reliant on early 2000s sources. Authors are encouraged to include and engage with more recent work, such as: Beugelsdijk, S., & Welzel, C. (2018). Dimensions and dynamics of national culture: Synthesizing Hofstede with Inglehart. Journal of Cross-Cultural Psychology, 49(10), 1469–1505. <https://doi.org/10.1177/0022022118798505> – discussing dimensions beyond I-C. Church, A. T. (2016). Personality traits across cultures. Current Opinion in Psychology, 8, 22–30. <https://doi.org/10.1016/j.copsyc.2015.09.014> – examining Big Five validity across cultures. Vignoles, V. L., Owe, E., Becker, M., Smith, P. B., Easterbrook, M. J., Brown, R., ... & Bond, M. H. (2016). Beyond the ‘east–west’ dichotomy: Global variation in cultural models of selfhood. Journal of Experimental Psychology: General, 145(8), 966–1000. <https://doi.org/10.1037/xge0000175> (already mentioned). | Thank you for your suggestions. I decided to keep to the Bonferroni correction to be more conservative, given the heterogeneity of the sample. I reported them in my paper accordingly (on p. 13 and p. 15).  Thank you for the suggested reading. I have incorporated them into my article accordingly. I excluded Church’s (2016) paper since I removed personality traits from my analysis. |
| 4. Minor Issues • Table formatting: several tables are overly dense and require clearer presentation (e.g., exact p-values, standardized effect sizes). • Language: minor grammatical issues persist – a native-level proofreading would benefit clarity and flow. • Reference formatting: Reference formatting should be checked for full compliance with PLOS ONE’s style guide. Although journal names are not italicized in this format, some inconsistencies are noticeable (e.g., missing volume numbers or inconsistent punctuation). Please ensure that all entries follow the required journal template. | Thank you for pointing this out. I have edited the paper accordingly. |
| 5. Recommendation While the study has potential and touches upon a meaningful and underexplored intersection between self-construals and personality, the issues above are non-trivial. They require both methodological and theoretical reconsideration. In this context, I suggest a thorough revision of the manuscript, addressing the following key points: • clear redefinition of group assignment criteria; • addressing low reliability metrics; • integrating updated theoretical perspectives; • correcting for multiple comparisons; • enhancing statistical reporting transparency. | Thank you for these recommendations. Based on your suggestions, I have adopted a different analytical strategy – i.e., multilevel regression analysis, nesting participants into regions instead of relying on a dichotomous grouping of collectivist vs. individualist (pp. 11-16). I removed the personality measures given the low reliability metrics. I have updated the theoretical perspectives (p. 8) and discussion (pp. 17-20) accordingly. I updated the statistical reporting (pp. 11-16) accordingly. |
