# Supplementary material for: “I feel good… I knew that I would…”: The role of self in musical reward across cultures
Source: PLoS One. 2026 Jan 6;21(1):e0340597. doi: 10.1371/journal.pone.0340597 (PMC12774338; doi:10.1371/journal.pone.0340597)
Supplement: Attachment — Submitted filename: ReviewerResponse_20251201.docx [file pone.0340597.s005.docx]

| **Reviewer 1 Comments** | **Author Response** |
| --- | --- |
| General Assessment  I appreciate the authors’ clear effort to substantially revise and refine this manuscript in light of prior feedback. The new version demonstrates both conceptual and methodological growth. In particular, the inclusion of a multifaceted self-construal measure (based on Vignoles et al., 2016, and the Y-SCS for the Chinese sample) marks a significant theoretical and empirical advancement. This addition allows for a far more nuanced understanding of self-related variation in musical reward and provides a welcome alternative to the earlier binary conceptualization (independent vs. interdependent). The revised analyses are pertinent, carefully interpreted, and better aligned with the study’s stated goals.  Overall, the manuscript now presents a richer and more theoretically coherent contribution to the emerging intersection between cultural psychology and music cognition. The authors’ responses to earlier critiques were thoughtful and, in most cases, convincingly addressed. | Thank you for your prior feedback; they were very helpful in refining this article further. |
| Major Issue for Clarification The only serious reservation I still hold concerns the temporal and procedural sequence related to the adoption of the new self-construal instrument. According to the Methods section (p. 17), the authors acknowledge that they obtained the multifaceted self-construal scale from Dr. Viv Vignoles after their initial correspondence (Aug 22, 2022) and receipt of the materials on Nov 15, 2022. However, data collection reportedly began on Nov 3, 2022—almost two weeks earlier. This raises an important question of study design integrity: - Was data collection initiated before the complete set of study instruments and design decisions were finalized? - If so, how was the new self-construal measure incorporated—was it added mid-study, or were some participants excluded and recollected? - If these data were indeed available at the time of the first submission, why were results from this more appropriate instrument not reported earlier? Clarifying this procedural timeline is crucial, because it directly affects the transparency and replicability of the research. The issue does not necessarily invalidate the findings, but it requires an explicit methodological statement in the manuscript (e.g., under “Limitations” or “Procedure”) to ensure that readers and editors understand how the final dataset corresponds to the revised study design. | Thank you for pointing this out. To clarify, data collection began after the complete set of study instruments were finalized. I have tripled-checked my data records to ensure that this is true. Ethical approval was received on 3 November 2022 with an expected project end date of 7 October 2023 – this was originally reported in the paper. Data collection only began after the study materials were finalized, with the first participant recruited on 27 November 2022 and the last participant on 25 March 2023. I have edited the text to reflect this timeline more accurately on p. 9.  This data was indeed available at the time of the first submission. In full disclosure, this is not the first journal that I submitted this paper to. I had considered the feedback from the other reviewers (and rejections) from previous journals, which meant that this paper has undergone several ‘evolutions.’ So, that is probably why it was not reported earlier. |
| Minor Comments and Suggestions 1. Methodological transparency: Please indicate explicitly (a) whether the full sample (N = 435) completed the multifaceted self-construal measure, and (b) whether any earlier data were discarded or replaced when adopting the new instrument. | Based on the timeline reported above, the full sample completed the 8-dimensional self-construal measure. |
| 2. Theoretical framing: The integration of the eight-dimensional self model could be strengthened by explicitly situating each dimension vis-à-vis the observed musical reward subtypes—for instance, noting how “self-direction vs. heteronomy” aligns with the musical seeking and mood regulation factors. | I’m not sure if I understand this. Did you mean “self-containment vs. connectedness to others” rather than “self-direction vs. reception to influence”? Also, are you referring to the way I reported the results on p. 16? I think it makes more logical sense to report the dimensions (Y-SCS) that explain the outcome (musical reward subtype), rather than multiple outcomes associated with specific dimensions. The latter seems confusing in terms of the logic. In addition, the former would be consistent with my earlier reporting on p. 14. For the purposes of comparing between musical reward subtypes, I have changed the table to a summary table on p. 17 as per your next suggestion. |
| 3. Statistical reporting: Although regression results are clearly reported, the presentation could be further improved by including a single summary table (perhaps in the Supplementary Material) that lists standardized β coefficients for all eight self-construal dimensions across the musical reward subtypes, for easier comparison. | Thank you for this suggestion. I agree with you that this makes it clearer to see the similarities and differences across the musical reward subtypes. I have included this summary table on p. 17 and shifted the full results table to the supporting materials. |

| **Reviewer 2 Comments** | **Author Response** |
| --- | --- |
| Thank you for the opportunity to review the article entitled ‘"I feel good... I knew that I would...": The role of self in musical reward across cultures’, submitted to the PLOS ONE. This study explores cultural differences in various types of musical reward experiences. It takes a novel approach by defining culture in terms of self-construal rather than nationality or the individualism–collectivism distinction based on nationality. The findings partially support the hypotheses, revealing different associations between music-related reward types and interdependent versus independent self-construal. Overall, I found the manuscript engaging, well-written, and suitable for publication. The introduction clearly leads into the research question and situates it within the relevant literature. The methods and results are well presented, and the discussion offers nuanced interpretations and thoughtful suggestions for future work. I also reviewed the previous reviewer’s comments and the authors’ revisions. I have only a few minor suggestions that may help strengthen the manuscript. | Thank you for taking the time to review this article and for your constructive comments. |
| 1. Hypotheses: It was not entirely clear to me why independent self-construal was expected to correlate with specific BMRQ subscales (musical seeking, emotion evocation, mood regulation, and sensory-motor rewards). The rationale for these particular predictions did not seem fully grounded in prior literature. For instance, as you note in the discussion, emotional responses to music can involve both self-focused emotions (e.g., feeling strong, secure, happy) and more social or relational emotions (e.g., awe, transcendence). Similarly, mood regulation through music may include socially oriented processes such as reducing loneliness or engaging in parasocial interaction (e.g., Bannister et al., 2025, Music & Science). I suggest clarifying the theoretical basis for these expectations; or, if more appropriate, addressing this literature in the discussion or future directions. | The predictions regarding independent self-construal were based on previous research on the uses and functions of music (discussed on p. 5). For example, members of individualistic societies tend to use music for self-centric purposes such as emotion regulation. I have included this clarification on p. 8 and the relevant references.  Thank you for suggesting the paper by Bannister et al. (2025). I agree that this is very relevant to my arguments. Per your suggestion, I have included this in the discussion on p. 19. |
| 2. Personality measure (response to reviewer 1 Based on the previous review, I understand that a personality measure and its associated analyses were initially included, but then removed due to low internal consistency. I agree that excluding the scale is appropriate in this case. However, for transparency, it may be helpful to add a brief explanation (even as a footnote) about why the measure was initially included and ultimately omitted. | Thank you for this suggestion. I have included a short description of the personality measure, why it was initially included, and why it was subsequently omitted on p. 11. |
| 3. Post-hoc power analysis: Thank you for adding the requested power analysis. However, it would be important to justify the expected effect size. | Thank you for your feedback. I am hesitant to do so because it presents almost a circular logic because it is a post-hoc power analysis, as opposed to an a priori power analysis. Nevertheless, I have added a justification on p. 16, which helps to tie the different analyses together. |
